# Computational Prediction of *O*-linked Glycosylation Sites That Preferentially Map on Intrinsically Disordered Regions of Extracellular Proteins

**DOI:** 10.3390/ijms11124991

**Published:** 2010-12-03

**Authors:** Ikuko Nishikawa, Yukiko Nakajima, Masahiro Ito, Satoshi Fukuchi, Keiichi Homma, Ken Nishikawa

**Affiliations:** 1 College of Information Science and Engineering, Ritsumeikan University/Noji-higashi 1-1-1, Kusatsu, Shiga 525-8577, Japan; E-Mail: nakajima.yukiko@gmail.com; 2 College of Life Sciences, Ritsumeikan University/Noji-higashi 1-1-1, Kusatsu, Shiga 525-8577, Japan; E-Mail: maito@sk.ritsumei.ac.jp; 3 Center for Information Biology & DNA Data Bank of Japan, National Institute of Genetics/Yata 1111, Mishima, Shizuoka 411-8540, Japan; E-Mails: sfukuchi@genes.nig.ac.jp (S.F.); khomma@lab.nig.ac.jp (K.H.); 4 Department of Bioinformatics, Maebashi Institute of Technology/Kamisadori 460-1, Maebashi, Gunma 371-0816, Japan; E-Mail: mit-nishikawa@maebashi-it.ac.jp

**Keywords:** protein *O*-glycosylation, mucin-type, posttranslational modification, support vector machine, clustered and isolated glycosylation sites, intrinsically disordered, extracellular protein, non-conservation property

## Abstract

*O*-glycosylation of mammalian proteins is one of the important posttranslational modifications. We applied a support vector machine (SVM) to predict whether Ser or Thr is glycosylated, in order to elucidate the *O*-glycosylation mechanism. *O*-glycosylated sites were often found clustered along the sequence, whereas other sites were located sporadically. Therefore, we developed two types of SVMs for predicting clustered and isolated sites separately. We found that the amino acid composition was effective for predicting the clustered type, whereas the site-specific algorithm was effective for the isolated type. The highest prediction accuracy for the clustered type was 74%, while that for the isolated type was 79%. The existence frequency of amino acids around the *O*-glycosylation sites was different in the two types: namely, Pro, Val and Ala had high existence probabilities at each specific position relative to a glycosylation site, especially for the isolated type. Independent component analyses for the amino acid sequences around *O*-glycosylation sites showed the position-specific existences of the identified amino acids as independent components. The *O*-glycosylation sites were preferentially located within intrinsically disordered regions of extracellular proteins: particularly, more than 90% of the clustered *O*-GalNAc glycosylation sites were observed in intrinsically disordered regions. This feature could be the key for understanding the non-conservation property of *O*-glycosylation, and its role in functional diversity and structural stability.

## 1. Introduction

Glycan, a carbohydrate chain, is considered the third life chain after DNA and protein [1]. Glycans bind to proteins or lipids, and more than 50% of the mammalian proteins are glycosylated [2] to acquire structural stability and function as well as the biodiversity of organisms. Abnormal carbohydrate chain modification occurs in several serious diseases such as familial tumoral calcinosis [3,4], Tn syndrome [5,6], IgA nephropathy [7–9], coronary artery disease [10,11], and tumor formation and metastasis [12–14].

The two major types of protein glycosylation in eukaryotes are *N*-linked and *O*-linked glycosylation. *N*-linked glycans are attached to the amide nitrogens of asparagine (Asn) side chains in the consensus sequences Asn-Xaa-Ser or Asn-Xaa-Thr, where Xaa represents any amino acid residue except proline (Pro) [15,16]. *O*-linked glycans are attached to the hydroxyl group of serine (Ser) or threonine (Thr) side chains [17]. *O*-linked glycosylation (*O*-glycosylation) encompasses several different types of glycosylation, such as *O*-GalNAc, *O*-GlcNAc, *O*-Fuc, *O*-Glc, *O*-Man, and *O*-Xyl glycosylation. In eukaryotes, the most common *O*-glycosylation is *O*-GalNAc glycosylation, or mucin-type *O*-glycosylation. In the mucin-type *O*-glycosylation, not all Ser or Thr residues are glycosylated, and no specific consensus sequence has been identified so far. One characteristic of the mucin-type *O*-glycosylations is the formation of clusters within repeated amino acid sequences, termed tandem repeats, which are rich in Ser or Thr residues [18–20]. Many glycoproteins contain one or more mucin-like domains, typically rich in Pro, Ser, and Thr residues, producing discrete regions in the entire molecule that are heavily decorated with mucin-type *O*-glycosylations [21].

On the basis of statistical analysis of mucin-type *O*-glycosylation sites and data on GalNAc-T (*N*-acetylgalactosamine transferase), the following general rules apply regarding mucin-type *O*-glycosylation [22]: (1) it is tissue specific (there are different GalNAc-T with overlapping but different specificities, and these GalNAc-T have different tissue-specific expression patterns); (2) it is mainly a post-translational and postfolding event (therefore, only surface-exposed Ser and Thr residues are glycosylated); and (3) it shows a primary sequence preference, which is different for Ser and Thr (Thr appears to be glycosylated more efficiently than Ser). Moreover, in a previous analysis of the structural context of mucin-type *O*-glycosylation sites by using the structural information on amino acid sequences of mucin-type *O*-glycoprotein from the Protein Data Bank (PDB) [23], 14 of 86 protein sequences were represented by structures in PDB. Of these 14 structures, two were represented twice in all 12 non-redundant structures. All sites were found in coil or turn regions located near the N- or C-termini of the protein, in linker regions between domains, or in coil regions connecting secondary structure elements. Ser and Thr residues annotated as mucin-type *O*-glycosylation are less likely to be precisely conserved among mammalian protein homologs and more likely to be surface-exposed than Ser or Thr residues without this annotation.

Regarding non-mucin-type *O*-glycosylation, *O*-GlcNAc-type glycosylation has recently attracted attention; it modifies eukaryotic nuclear and cytosolic proteins and is as dynamic and possibly as abundant as Ser or Thr phosphorylation. *O*-GlcNAc glycans are attached to the hydroxyl group of Ser or Thr residues. The functions of *O*-GlcNAc proteins are known for cytoskeletal proteins and their regulatory proteins, such as viral proteins, nuclear-pore and nuclear-oncogene proteins, RNA polymerase II catalytic subunit, and numerous transcription factors. Despite their functional diversity, all these proteins are also phosphoproteins [24].

Proteins with partially or fully intrinsically disordered (ID) structures have been well investigated in the past few years and are found mostly in eukaryotes. They are frequently involved in key biological processes such as cell cycle control, transcriptional and translational regulation, membrane fusion and transport, and signal transduction [25–28]. Several characteristics of ID structures have been elucidated [29,30]: (1) sequence repetitions consisting of a shorter sequence pattern are often contained in ID regions [31]; (2) sequence conservation is extremely poor in ID, because ID regions have higher evolution rates than structural domains [32]; (3) most ID regions exist in linkers connecting domains and/or in terminal tails, but some are inserted in structural domains [33]; (4) the frequency of intrinsically disordered proteins (IDPs) is higher in eukaryotes than in prokaryotes [34]; (5) most IDPs localize to the nucleus [34]; and (6) protein phosphorylation, another type of post-translational modification, predominantly occurs in ID regions [35]. Currently, some of these characteristics are known to be similar to *O*-glycosylation.

In this study, to elucidate the *O*-glycosylation mechanism, we first applied a support vector machine (SVM) [36] to predict whether a Ser or Thr residue is glycosylated. Similar statistical machine learning approaches for the prediction of the mucin-type *O*-glycosylation site of have been reported [23,37]. A pioneering work by Julenius *et al*. [23] used a layered neural network for prediction. The results led to the conclusion that the bulk properties are the main factor for *O*-glycosylation, as bulk average properties including amino acid composition gave the best prediction. There are other reports which pointed out the position specific properties of amino acids around *O*-glycosylation sites: for example, high existence ratios of proline (Pro) at −1 and +3 relative to *O*-glycosylation sites [38]. One of the objectives of this study was to identify the crucial properties of the protein for the sites to be *O*-glycosylated, based on performance of machine learning. *O*-glycosylated sites were often found clustered along the sequence, whereas others sites were located sporadically. Therefore, we chose the strategy to classify the *O*-glycosylation sites into two types, *i.e.*, the clustered and isolated types, and to separately determine the essential properties, and see if they differ from each other. We obtained mammalian protein sequence data with *O*-glycosylation site information from UniProt [39], developed two types of SVMs for predicting clustered and isolated sites separately, and calculated the existence frequencies of amino acids around *O*-glycosylation sites for the two types to estimate the existence probabilities of amino acids at each position relative to the glycosylation sites. We also conducted an independent component analysis (ICA) of the amino acid sequences to elucidate whether the position-specific existences are independent. Finally, we found that *O*-glycosylation is preferentially located within ID regions of extracellular proteins. So far as we are aware, no reports have hitherto discussed *O*-glycosylation in relation to ID regions or IDPs.

## 2. Results

### 2.1. Prediction by SVM

**Figure 1.**
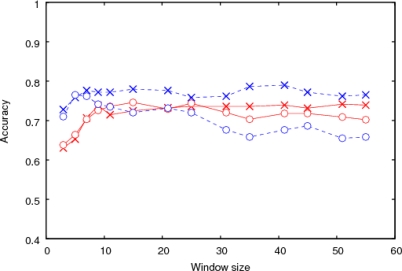
Prediction accuracies for the clustered and isolated types of mucin-type *O*-glycosylation in various sequences varying in length (window size, *W*_s_) from three to 55. Amino acid sequence or composition information was used as the input to SVM. The crosses and circles indicate the prediction accuracies obtained by using the sequence information and composition information, respectively. The clustered and isolated types are shown in red and blue, respectively.

SVM was trained for each clustered or isolated type of mucin-type *O*-glycosylation separately. The exact definitions of the clustered and isolated types of *O*-glycosylations are given in Section 4.2. The input to SVM was information on a protein sequence of a fixed length including the prediction target site at the center. Two types of information were used: one was the amino acid sequence encoded by sparse coding, which distinguished all 20 types of amino acids, while the other was the amino acid composition of the sequence. Figure 1 shows the prediction accuracy obtained by using either sequence or composition information as the input to SVM for the clustered or isolated type of *O*-glycosylation.

First, we focused on the results obtained by using sequence information. The prediction accuracy for the clustered type increased according to the window size (*W*_s_) up to about *W*_s_ = 31, with the highest value 74% obtained at *W*_s_ = 51. On the other hand, for the isolated type, the accuracy remained almost constant, including at *W*_s_ = 3. The highest accuracy was 79% obtained at *W*_s_ = 41. Therefore, the sequence information up to the 15th nearest neighbor was effective for predicting clustered glycosylation, and isolated glycosylation was primarily affected by closer neighbors.

Next, we compared the results of the sequence information analysis with those obtained using composition information. For the clustered type, the accuracy and *W*_s_ dependency with the composition information were similar to those with the sequence information. However, for the isolated type, the accuracy decreased according to *W*_s_ when the input was composition.

**Figure 2.**
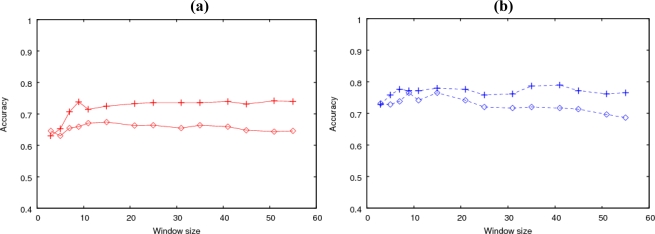
(**a**) Prediction accuracies of the two SVMs for the clustered glycosylation. The crosses and circles represent the prediction obtained using the SVM trained by the clustered and isolated type, respectively. The input was the sequence information. **(b**) Prediction accuracies of the two SVMs for the isolated glycosylation. The crosses and circles represent the prediction obtained using the SVM trained by the isolated and clustered type, respectively.

The difference between the two types of trained SVMs was demonstrated by comparing their prediction accuracies for both clustered and isolated types. Figure 2(a),(b) shows the prediction accuracies using the two SVMs for the clustered and isolated types, respectively. The input was sequence information. From the results shown in Figure 2, each SVM was specialized for the type used in the training.

These results indicated that site-specific information of the amino acid residues was effective for predicting isolated glycosylation, whereas only the gross composition up to about the 15th neighbor affected clustered glycosylation.

### 2.2. Existence Frequency of Amino Acids around *O*-Glycosylation Sites

According to the previous results, it is likely that glycosylation biosynthesis between clustered and isolated types is different, and the site-specific existence of some amino acids affects the glycosylation, especially for the isolated type. Therefore, any motif can be expected from the combination of such amino acid existences.

**Figure 3.**
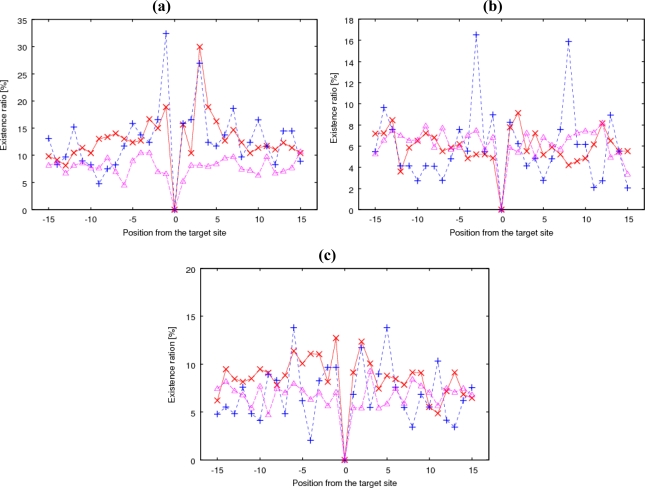
(**a**) Existence ratios of Pro at each position for clustered positive, isolated positive, and negative Ser or Thr sites (indicated by red crosses, blue crosses, and pink triangles, respectively). Existence ratios of Val (**b**) and Ala **(c)** shown in a similar style.

The existence ratio or probability was calculated for 20 types of amino acids at a relative position from the glycosylation site for clustered and isolated glycosylations separately. As a typical example of the results, Figure 3(a) shows the existence ratio of proline (Pro) at each relative position within *W*_s_ = 31 around the clustered positive, isolated positive, and negative Ser and Thr residues. Pro has a high ratio at −1 and +3 relative to the *O*-glycosylation site [38]. In the figure, the high peak can be noted at −1 only for isolated glycosylation and at +3 for both types. The peak at −1 leads to the high prediction accuracy even at *W*_s_ = 3 for the isolated type shown in Figure 1.

Similarly, for valine (Val), Figure 3(b) shows very sharp peaks of 16% both at −3 and +8 only for isolated glycosylation. Alanine (Ala) had peaks at −6 and +5, again only for isolated glycosylation (Figure 3(c)). On the contrary, cysteine (Cys) residues were rarely observed near clustered glycosylation sites. In summary, a high site-specific existence of certain amino acids was especially observed for isolated glycosylation.

### 2.3. Independence of the Amino Acid Existences

**Figure 4.**
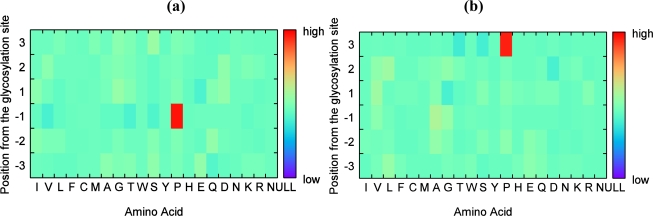
Independent components of the amino acid sequence around the isolated glycosylation sites, corresponding to the high existence probability of (**a**) Pro at −1, and (**b**) Pro at +3. The horizontal axis indicates 20 amino acids and a null, and the vertical axis indicates the relative position to a glycosylation site and ranges from −3 to +3. The gradation of each box shows the existence ratio of each amino acid at each position.

ICA was applied for the amino acid sequence around a glycosylated site to elucidate whether each amino acid existence correlates with or is independent of each other. Figure 4 shows two examples of the obtained independent components for the isolated type.

Figure 4 shows the well-known high probabilities of Pro at −1 and +3. Supplemental Figure S1 shows the newly found Val at −3 and Ala at +2 as independent components. This finding means that the amino acids exist in a certain position independently and affect isolated glycosylation. Other components also possess each high probability element. The probability of Pro was also high at +2, −2, −3, and +1 (shown in descending order of probability).

Figure S2 shows two components for the clustered glycosylation. Figure S2(a) shows that Pro at +3 had a remarkably high probability for this type as well. However, most components did not show high probability compared with those obtained for the isolated type (Figure S2(b)).

### 2.4. Occurrence of O-GalNAc Glycosylation in Domains or Intrinsically Disordered Regions

Structural domains and ID regions of mucin-type *O*-glycoproteins (*O*-GalNAc) were analyzed using DICHOT [40,41] to determine the relationship between *O*-glycosylation and ID regions. All residues were binary classified into structural domains and ID regions. Further, 107 mammalian mucin-type *O*-glycoproteins were taken from the UniProt database (Release 14.0). The results of DICHOT were directly downloadable for 62 human proteins, whereas those for 45 non-human proteins were newly calculated. Table 1 shows the frequencies of *O*-glycosylation in relation to ID regions. The total numbers of all amino acid residues in the 107 proteins and their ID regions were 45,962 and 14,028, respectively. Thus, the existence ratio of ID regions in the 107 *O*-glycoproteins was 30.5% (14,028/45,962). On the other hand, the numbers of all *O*-glycosylation sites in the 107 *O*-glycoproteins and *O*-glycosylation sites in their ID regions were 465 and 399, respectively. Thus, the existence ratio of ID regions in the *O*-glycosylated sites was 85.8% (399/465).

The existence ratio of *O*-glycosylation sites in ID regions was 2.84% (399/14,028), which was substantially higher (2.8-fold) than that of *O*-glycosylation sites in the 107 *O*-glycoproteins (465/45,962 = 1.01%). On the contrary, the existence ratio of *O*-glycosylation sites in structural domains was 0.21% (66/31,934).

When we calculated the *O*-glycosylation ratio over Ser and Thr sites as a reference, the total numbers of Ser and Thr residues in the 107 proteins and their ID regions were 7228 and 2779, respectively. Thus, the *O*-glycosylation ratio in ID regions was 14.4% (399/2779), which was substantially higher (2.2-fold) than that in the 107 *O*-glycoproteins (465/7228 = 6.43%). On the contrary, the *O*-glycosylation ratio in structural domains was 1.48% (66/4449).

When we distinguished between clustered and isolated *O*-glycosylation sites, there were 283 (2.02%) and 116 (0.83%) sites, respectively, in ID regions. The existence ratios of ID regions in clustered and isolated *O*-glycosylated sites were 91.0% and 75.3%, respectively.

In brief, *O*-glycosylation occurs more frequently in ID regions than in structural domains, and this tendency is more remarkable for clustered glycosylation.

**Figure 5.**
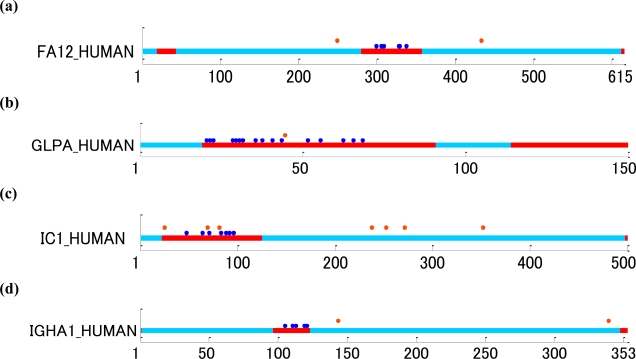
Glycosylation sites plotted along with the distinction between structural domains and ID regions of human glycoproteins. The light blue and red regions correspond to structural domains and ID regions, respectively, and the blue and orange dots indicate mucin-type *O*-linked (GalNAc) and *N*-linked sites, respectively. (**a**) FA12_HUMAN: coagulation factor XII with *O*-linked (GalNAc) modifications at T299, T305, S308, T328, T329 and T337, and *N*-linked (GlcNAc) modifications at N249 and N433. (**b**) GLPA_HUMAN: glycophorin-A with *O*-linked sites at S21, T22, T23, T29, S30, T31, S32, T36, S38, S41, T44, T52, T56, S63, S66 and T69, and *N*-linked site at N45. (**c**) IC1_HUMAN: plasma protease C1 inhibitor with *O*-linked sites at T48, S64, T71, T83, T88, T92 and T96, and *N*-linked sites at N25, N69, N81, N238, N253, N272 and N352. (**d**) IGHA1_HUMAN: Ig α-1 chain C region with *O*-linked sites at S105, S111, S113, S119 and S121, and *N*-linked sites at N144 and N340.

Figure 5 shows examples of mucin-type *O*-glycoproteins. Six sites of coagulation factor XII (UniProt ID: FA12_HUMAN) of secreted protein [42] were modified by mucin-type *O*-glycosylation. In addition, glycophorin-A (UniProt ID: GLPA_HUMAN) of cell membrane protein [43,44], plasma protease C1 inhibitor (UniProt ID: IC1_HUMAN) of secreted protein [45], and Ig α-1 chain C region (UniProt ID: IGHA1_HUMAN) of immunoglobulin were *O*-glycosylated at 16, seven, and five sites, respectively. The results of 62 human proteins are shown in Supplemental Figure S3.

These results support the hypothesis that many mucin-type *O*-glycoproteins are glycosylated for clustered modifications as clusters in ID regions. This is based on the examination of individual cases revealing the clustering of most mucin-type *O*-glycoproteins in ID regions (Figures 5 and S3) and nearly all of the small number of the clustered mucin-type *O*-glycosylation sites in structural domains (Figure S3) were very close to the boundary with ID regions. CSF2_HUMAN serves as an example of the latter case (Figure S3): *O*-GalNAc is added to 27 Thr located in a structural domain, but right next to the ID region that extends to residue 26 (Figure S3). Furthermore, most of the isolated mucin-type *O*-glycosylation sites also fell in ID regions (Figure S3), while a majority of those in structural domains were located in loop regions (e.g., the *O*-glycosylation site in APOH_HUMAN, Figure S3).

### 2.5. Non-Mucin-Type *O*-Glycosylation

Eighty-three non-mucin-type glycoproteins, including those with *O*-GlcNAc, *O*-Gal, *O*-Xly, *O*-Fuc, *O*-Glc, *O*-HexNAc, and *O*-Hex modifications, were collected from UniProt database 14.0. ID regions of these glycoproteins were again identified using DICHOT [40,41]. The existence ratios of *O*-GlcNAc (78.9%) and *O*-Xly (85.0%) were high in ID regions (Table 2). This high ratio was caused by the high Ser or Thr ratio in ID regions for *O*-GlcNAc (77.1%), whereas *O*-glycosylation preferentially occurred in ID regions for *O*-Xly, similar to that for *O*-GalNAc glycosylation.

Clustered *O*-glycosylation sites were rarely found in the non-mucin-type, compared with the mucin-type. One rare example was the clustered *O*-Xly glycosylation sites in SRGN_HUMAN.

The ratio of Ser or Thr residues for glycosylation was remarkably high in the mucin-type in ID regions (399/2779 = 14.4%), compared with the non-mucin-type.

## 3. Discussion

According to the finding that *O*-GalNAc glycosylation sites often clustered along the sequence, we classified the *O*-glycosylation sites into clustered and isolated types using a simple criterion. The SVM prediction indicated that the amino acid composition was effective for the clustered type, whereas the site-specific algorithm was effective for the isolated type. The highest prediction accuracy of the clustered type was 74%, while that of the isolated type was 79%. Moreover, more than 90% of the clustered *O*-GalNAc glycosylation sites were located in ID regions. In the isolated type, some amino acid residues were observed at high frequencies at certain positions relative to *O*-glycosylation sites (Pro at −1 and +3, Val at −3 and +8, and Ala at −6 and +5). Addition of ID region information to the SVM input improved the prediction accuracy only slightly, implying that amino acid composition as an input to an SVM provides most information on ID propensity.

Previously [23], *O*-GalNAc glycosylation sites were predicted by using a layered neural network; this study indicated that bulk average properties including amino acid composition give the best prediction. This is the property of clustered glycosylation sites, which constitutes the majority of *O*-GalNAc glycosylation sites (Table 1). The non-conservation of glycosylation sites they discussed is the result of a high fraction of ID regions and generally low conservation of ID regions [40]. In another report [38], *O*-glycosylation sites were classified into multiple and isolated types, roughly corresponding to the clustered and isolated types, respectively, in this paper. However, their criterion differed from ours: *O*-glycosylation sites were defined as multiple when there was at least one more glycosylation site within the tenth-nearest neighbor. They found high frequencies of Ser and Thr around multiple glycosylation sites, and proposed the use of this property for predicting the multiple type. Further, they indicated high site-specific frequencies of Pro at −1 and +3 for the isolated type, which is consistent with the present results. However, they concluded that the finding was not sufficiently useful for prediction and did not consider ID regions.

The four examples of *O*-GalNAc glycosylation (Figure 5) illustrate the high frequency of *O*-GalNAc glycosylation sites in ID regions: all of the *O*-linked glycosylation sites in the figure belonging to both clustered and isolated types fall in ID regions identified by the DICHOT system. In predicting the clustered type of *O*-GalNAc glycosylation, the amino acid composition near the sites was more effective than sequence information (Figure 1). Interestingly, this type of *O*-linked glycosylation is mostly located in ID regions and rarely in structural domains (Table 1). As ID regions generally have a skewed amino acid composition without specific sequence characteristics [33,46], the current finding makes sense because the characteristic amino acid composition of ID regions is likely to be a good predictor of the clustered type of *O*-linked glycosylation. The finding that addition of ID region information to SVM input does not drastically improve the accuracy of prediction supports this idea.

For the isolated type of *O*-GalNAc glycosylation, however, amino acid sequence information is a better predictor than amino acid composition (Figure 1). In this type, 75% of the sites fall into ID regions, much higher than the average fraction of ID regions in *O*-GalNAc glycosylated proteins (30.5%), while the remaining 25% are located in structural domains (Table 1). The small but significant fraction in structural domains partially explains the sequence finding: certain sequence characteristics are needed for this type of *O*-linked glycosylation site to be located at the molecular surface. The finding that Pro at −1 and +3 occur at high frequency indicates that Pro working as a breaker of α-helix and β-sheet is important for the site to accommodate *O*-GalNAc glycosylation. *O*-GalNAc glycosylation sites of the isolated type are also often found in ID regions very close to the boundary of structural domains. In such cases too, sequence characteristics in the vicinity are likely to be crucial for making the sites available to *O*-linked glycosylation.

In both mucin (*O*-GalNAc) and non-mucin types of *O*-linked glycosylation (*O*-GlcNAc and all the rest), *O*-glycosylation occurs post-translationally (*i.e*., after protein folding) [22]. In this discussion, we first limited our attention to mucin-type and the three most prevalent non-mucin-type *O*-linked glycosylations, namely *O*-GlcNAc, *O*-Gal, and *O*-Xyl (Table 2). The table shows that ID regions are generally preferred irrespective of the types of *O*-linked glycosylation, consistent with the view that enzymes that add these types of *O*-linked glycosylation recognize structural features of proteins. Quite possibly, *O*-linked glycans of these types are attached to residues in ID regions to prevent protease degradation of glycosylated proteins. Increased *O*-GlcNAc modification of human RNA polymerase II transcription factor SP1, for instance, deters its degradation in the proteasome [47] and *O*-GalNAc modification of the human CD44 antigen inhibits cleavage of the extracellular domain by specific proteases [48]. On the other hand, two other non-mucin types of *O*-glycosylations of *O*-Fuc and *O*-Glc occur in domain regions. *O*-Fuc and *O*-Glc have been found on epidermal growth factor (EGF)-protein domains and have consensus sequences. *O*-Fuc is attached to the Thr or Ser residue in -Cys-Xaa-Xaa-Gly-Gly-Thr/Ser-Cys- [49], and *O*-Glc is attached to the Ser residue in -Cys-Xaa-Ser-Xaa-Pro-Cys- [49].

The subcellular localizations and consequently cellular functions of *O*-GalNAc and *O*-GlcNAc are quite different: *O*-GalNAc is added to proteins sometimes in the endoplasmic reticulum, but mostly in the Golgi apparatus [50]. The modified proteins become either extracellular proteins or plasma membrane proteins with the *O*-GalNAc glycosylation sites in the extracellular domains. *O*-GalNAc glycosylation is tissue specific, because different GalNAc-Ts with overlapping but different specificities exist and have distinct tissue-specific expression patterns [51]. In contrast, *O*-GlcNAc glycosylation is a reversible modification of cytoplasmic and nuclear proteins and plays a regulatory role in competition with phosphorylation in some proteins [35,52,53]. Naturally, the biological significance of *O*-GalNAc glycosylation is distinct from that of *O*-GlcNAc. *O*-GalNAc modification affects extracellular processes such as cell adhesion, immunological recognition, and secretion [22], while *O*-GlcNAc modification is involved in transcription regulation, protein trafficking and turnover, among others, with a complex dynamic interplay with phosphorylation [54]. It will be interesting to investigate how prevention of protein degradation by *O*-linked glycosylation in ID regions is involved in various biological functions.

## 4. Materials and Methods

### 4.1. Protein Data Sets

The experimentally validated *O*-glycosylated Ser and Thr residues in mammalian proteins were selected from the UniProt database (Release 12.2) for the analysis in Sections 2.1–2.3. Ninety-eight proteins were obtained by annotation of mucin-type *O*-glycosylation by excluding “potentially,” “probably,” and “by similarity” annotations. There were 452 annotated Ser and Thr sites, and 6004 Ser and Thr sites without annotation, which were denoted positive and negative sites, respectively. Further, there were several homologs among the 98 proteins. Therefore, as a preliminary analysis, we examined whether the existence of these homologs affects the SVM-based prediction by selecting only one protein among the homologs with a similarity threshold down to 0.2. This step did not largely change the prediction accuracy obtained by ten-fold cross validation (Section 4.3). Therefore, all 98 proteins were used in the study.

The protein data used for the analysis in Sections 2.4 and 2.5 were obtained from the UniProt database (Release 14.0). One hundred and seven proteins were obtained as showing mucin-type *O*-glycosylation (*O*-GalNAc) and 83 proteins were obtained as showing non-mucin-type *O*-glycosylation of *O*-GlcNAc, *O*-Gal, *O*-Xly, *O*-Fuc, *O*-Glc, *O*-HexNAc, and *O*-Hex (Table 2). Among the 107 proteins with mucin-type *O*-glycosylation, 62 were human and 45 were non-human. Among the 83 proteins with non-mucin-type *O*-glycosylation, 38 were human and 45 were non-human.

### 4.2. Clustered and Isolated *O*-Glycosylation Sites

**Figure 6.**
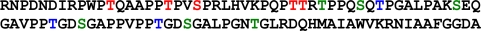
Example of clustered and isolated *O*-glycosylation sites. Ser or Thr residues of clustered, isolated, and of non-glycosylated sites, are indicated in red, blue and green, respectively.

Many positive sites were densely clustered, whereas others were located sporadically. We defined the two types of positive sites as follows: if the nearest neighbor Ser or Thr site on either side was glycosylated, it was termed a clustered *O*-glycosylation site; otherwise, it was considered isolated (Figure 6). Accordingly, among the 452 positive sites, 307 were clustered and the remaining were isolated. Glycoprotein MUCAP_PIG had the highest number of clustered sites, including 31 clustered modification sites among 1148 amino acids, and glycoprotein CEL_HUMAN had the highest number of isolated sites, including 10 isolated modification sites among 741 amino acids. These multiple isolated sites were caused by repeated segments of Thr-Gly-Asp-Ser, with glycosylated Thr and non- glycosylated Ser.

The SVM was trained for each type separately. In the predictions, all positive sites were used, and the same number of negative sites was randomly selected by uniform probability.

The input to SVM was a protein sequence including the prediction target site. A sequence of fixed length, *W*_s_, was excised from the original protein sequence with a prediction target of a Ser or Thr residue at the center. For example, a target site and the first to third nearest-neighbor amino acid residues on both sides constitute a sequence of *W*_s_ = 7. *W*_s_ varied from three to 55 in the predictions.

Two types of information on the *W*_s_ sequence were used as the input. One type of information was the amino acid sequence encoded by a sparse coding with 21 bits, which distinguished all 20 types of amino acids and a null (outside the protein terminal). Therefore, the amino acid sequence information was expressed as a 21(*W*_s_ − 1) +2 bits binary vector. The other input information was the amino acid composition, which was expressed as a 21-dimensional real value vector.

### 4.3. Prediction by SVM

Radial basis function was used as an SVM kernel, which was given by the following:

(1) $$\text{K}(\text{x},\;\text{x}\text{'})∼\text{exp}(-\gamma {\left\|\text{x}-\text{x}\text{'}\right\|}^{2})$$

where γ is the kernel parameter. Another parameter was margin size, denoted by *C*. We used the open software package SVM-light. *C* varied from 0.1 to 100 and γ varied from 1.0 × 10^−4^ to 1.0.

Ten-fold cross-validation was used for the learning and the evaluation to utilize the limited number of data fully. In this validation, each protein was grouped into one of 10 groups. Then, all positive sites and the same number of negative sites selected from the proteins in the group were used as training and validating samples. The performance was evaluated by the prediction accuracy averaged over 10 groups, and *C* and γ with the best performance were selected for each *W*_s_ value.

### 4.4. Amino Acid Sequence for ICA

ICA was applied for an amino acid sequence of *W*_s_ = 7 around the glycosylation site for the 307 clustered and 145 isolated sites. Six amino acids except the glycosylation site, regardless of whether it was Ser or Thr, were expressed by 21-bit sparse coding to form a 126-dimensional binary vector.

Then, principal component analysis (PCA) was used to reduce the dimensional size, and only the top 10 principal components were used for the ICA. Thus, 10 independent components were obtained for each type. As the original data were encoded by sparse coding, which directly indicates the existence of each amino acid, the value of the vector element corresponded to the existence ratio of each amino acid at a certain position.

### 4.5. Binary Prediction of Ordered/Disordered Protein Segments by DICHOT

Binary classification of protein molecules into structural domains and ID regions was performed by using the DICHOT system [40], which was applied to all the 107 proteins with mucin-type *O*-glycosylation and the 83 proteins with non-mucin-type *O*-glycosylation analyzed in this study. The DICHOT system assigns structural domains with similarity to known 3D structures by the method used in the GTOP database [54], which is a genome wide structural assignment database. The un-assigned regions in this process were judged with a combination of pre-existing and newly developed programs to discriminate structural domains and ID regions. Among the 107 proteins with mucin-type *O*-glycosylation, the results of DICHOT are available for the 62 human proteins at the web site [41], but not for the 45 non-human proteins. In addition, the revised result was used for one human protein, CEL_HUMAN, whose sequence length was changed because of an update of UniProt.

## 5. Conclusions

We found that the classification of mucin-type (*O*-GalNAc) glycosylation into clustered and isolated types is useful in developing algorithms to accurately predict *O*-GalNAc glycosylation sites. Furthermore, we discovered that most *O*-GalNAc and *O*-GlcNAc glycosylation sites are in ID regions. We propose that these *O*-linked glycans protect the ID regions from degradation and are crucial in controlling cellular functions.

## Figures and Tables

**Table 1.** Frequencies of occurrence of *O*-GalNAc glycosylations at clustered, isolated, and total glycosylation sites in ID regions. The total numbers of Ser or Thr residues, and the total numbers of amino acid residues are also shown for reference. 107 proteins were taken from UniProt 14.0, and ID regions were obtained from DICHOT [40,41].

		**Number of sites in ID**	**Total number of sites in 107 proteins**	**Ratio to be in ID (%)**
*O*-linked sites	Clustered	283	311	91.0
	Isolated	116	154	75.3
	Total	399	465	85.8
Ser/Thr sites		2,779	7,228	38.4
All sites in 107 proteins		14,028	45,962	30.5

**Table 2.** Frequencies of occurrence of the mucin type and non-mucin-type *O*-glycosylations at residue sites in ID regions. The total numbers of Ser and Thr residues are also shown for reference. 190 proteins were taken from UniProt 14.0, and ID regions were obtained from DICHOT [40,41].

***O*-linked type**	**Number of proteins**	**Number of sites in ID**		**Total number of sites in the proteins**		**Ratio to be in ID (%)**	
		***O*-linked**	**Ser/Thr**	***O*-linked**	**Ser/Thr**	***O*-linked**	**Ser/Thr**
*O*-GalNAc	107	399	2,779	465	7,228	85.8	38.4
*O*-GlcNAc	28	45	4,076	57	5,287	78.9	77.1
*O*-Gal	14	23	376	43	1,365	53.5	27.5
*O*-Xyl	20	34	649	40	1,593	85.0	40.7
*O*-Fuc	8	1	62	14	572	7.1	10.8
*O*-Glc	8	0	91	8	447	0	20.4
*O*-HexNAc*	4	3	94	4	136	75.0	69.1
*O*-Hex**	1	1	23	1	53	100.0	43.4

* *O*-HexNAc (*O*-GalNAc or *O*-GlcNAc)

** *O*-Hex (*O*-Gal or *O*-Glc)
